# Ghrelin misbalance affects mice embryo implantation and pregnancy success by uterine immune dysregulation and nitrosative stress

**DOI:** 10.3389/fendo.2023.1288779

**Published:** 2023-12-01

**Authors:** Eugenia Mercedes Luque, Cintia María Díaz-Luján, Daniela Andrea Paira, Nicolás de Loredo, Pedro Javier Torres, Verónica Inés Cantarelli, Ricardo Fretes, Rubén Darío Motrich, Ana Carolina Martini

**Affiliations:** ^1^ Instituto de Fisiología, Facultad de Ciencias Médicas, Universidad Nacional de Córdoba, Córdoba, Argentina; ^2^ Instituto de Investigaciones en Ciencias de la Salud (INICSA), Consejo Nacional de Investigaciones Científicas y Tecnológicas (CONICET)/Universidad Nacional de Córdoba (UNC), Córdoba, Argentina; ^3^ Instituto de Biología Celular, Facultad de Ciencias Médicas, Universidad Nacional de Córdoba, Córdoba, Argentina; ^4^ Departamento de Bioquímica Clínica, Facultad de Ciencias Químicas, Universidad Nacional de Córdoba, Córdoba, Argentina; ^5^ Centro de Investigaciones en Bioquímica Clínica e Inmunología (CIBICI), Consejo Nacional de Investigaciones Científicas y Tecnológicas (CONICET)/Universidad Nacional de Córdoba (UNC), Córdoba, Argentina

**Keywords:** (D-Lys3)GHRP-6, NOS, nitrosative stress, ILs, MMP9, NK cells, dendritic cells, T cells

## Abstract

**Introduction:**

In a previous study we found that ghrelin (Ghrl) misbalance during the peri-implantation period significantly impaired fetus development. In this study we aimed to evaluate the putative mechanisms underlying these effects, including embryo implantation success, uterine nitric oxide synthase (NOS) activity, nitric oxide synthesis and the inflammatory/immune uterine profile.

**Methods:**

Ghrelin misbalance was induced by injecting 4nmol/animal/day of Ghrl (hyperghrelinemia) or 6nmol/animal/day of a Ghrl antagonist (Ant: (D-Lys3)GHRP-6) from day 3 to 8 of pregnancy. Control animals (C) were injected with de vehicle. Females were euthanized at pregnancy day 8 and their uteri excised in order to evaluate: the percentage of reabsorbed embryos (microscopically), eNOS, iNOS and nytrotirosine expression (by immunohistochemistry), nitrite synthesis (by Griess technique), VEGF, IL-10, IL-17, IL-6, MMP9 and GM-CSF expression (by qPCR) and leukocyte infiltration by flow cytometry (evaluating T cells, NK cells, granulocytes, dendritic cells and macrophages).

**Results:**

Ant-treatment significantly increased the percentage of reabsorbed embryos and the uterine expression of eNOS, iNOS and nytrotirosine. (D-Lys3)GHRP-6-treatment increased also the expression of the inflammatory cytokines IL-6, IL-17 and MMP9, and decreased that of IL-10 (anti-inflammatory). Moreover, Ant-treatment increased also the NK cells population and that of CD11b^+^ dendritic cells; and decreased T cells percentages. Similarly, hyperghrelinemia showed a significant increase vs. C on eNOS, iNOS and nytrotirosineuterine expression and a decrease in T cells percentages.

**Conclusion:**

Ghrl misbalance during the peri-implantation period induces pro-inflammatory changes and nitrosative stress in the gravid uterus, impairing significantly embryo implantation and/or development.

## 1 Introduction

Ghrelin (Ghrl) is a 28 amino-acids gut peptide originally described by its orexigenic properties and its ability to stimulate growth hormone secretion (1). Nevertheless, since its discovery in 1999, Ghrl and its active receptor (GHS-R1a) has been described as ubiquitously expressed, reporting for the peptide several endocrine, paracrine and metabolic functions (2).

In several mammalian species, it has been shown that natural plasma Ghrl levels increase in dams as well as in fetuses, which supports the hypothesis that the peptide exerts a physiological role on gestation (3–5). Indeed, Ghrl has been proposed to modulate embryo implantation for several reasons: 1) mammalian endometrium and placenta express GHS-R1a (6–8), 2) in humans, cells from the endometrial epithelium, endometrial stroma, glandular epithelium and extravillous trophoblast synthesize Ghrl, showing a prominent increase in its expression during the first trimester of pregnancy (7, 8), and 3) *in vitro* experiments have shown a decidualization-stimulating effect of Ghrl upon endometrial stromal cells (7, 8). A central feature is that these stimulating effects of Ghrl take place at physiological levels (9–11). However, at high concentrations the peptide may operate as a food scarcity signal exerting inhibitory effects on reproduction, with the supposed aim of prioritizing life (12, 13). Supporting evidence reported by Sabatini et al. (2009) showed that infertile women presenting hyperghrelinemia, when undergoing assisted reproduction exhibited reduced clinical pregnancy outcomes (14).

Besides decidualization, Ghrl has also been involved in placental development and remodeling. It has been shown that *in vitro* the peptide stimulates proliferation, and decreases apoptosis, of the placental cell line JEG-3 (15). Moreover, diverse angiogenic properties have also been described for Ghrl (16, 17). Evidences from non-reproductive experimental models have shown that Ghrl enhances nitric oxide synthase (NOS) activity and, consequently, nitric oxide (NO) production (18–20). Nitric oxide is known to play a role in embryo implantation, placental development and nutrient transport (21–24). In fact, Kulandavelu et al. (2012) reported that endothelial NOS (eNOS) knock-out mice show decreased utero-placental blood flow, reduced uterine artery diameter and decreased spiral artery length (21). Furthermore, Kusinski et al. (2012) showed that fetuses from eNOS knock-out dams display fetal growth restriction, evidenced by significant reduction in body weight and abdominal circumference (22). In line with those findings, GHS-R1a knock-out mice (*Ghsr* ^-^/^-^) show reduced vasculature in adipose tissue (17). All these data suggest that abnormal ghrelinemia might impair embryo implantation, placentation and/or fetal growth. Nevertheless, scarce to absent data has been reported on that regard demonstrating that the role of hypo/hyperghrelinemia on embryo implantation and reproductive success has been scarcely studied.

On the other hand, Ghrl has been shown to be involved in other processes that might also impact early gestation. In fact, Ghrl has been shown to exert potent anti-inflammatory and immunomodulatory effects, which are key for the achievement of a successful pregnancy (25–27). The expression of the GHS-R1a in various lymphoid organs, T and B cells, monocytes and dendritic cells has already been described (reviewed in 25). Interestingly, T cells constitutively express the GHS-R, which significantly increases upon cell activation (28). In addition, B cells, monocytes and dendritic cells can secrete important amounts (ng) of Ghrl upon activation (25). Furthermore, T cells exposed to Ghrl show reduced expression of inflammatory cytokines (i.e. IL-1β, IL-6 and TNF-α), decreased proliferation and response, enhanced expression of immunoregulatory cytokines (i.e. IL-10) and induction of regulatory T cells (Treg) (25, 27, 29). Noteworthy, Ghrl or its agonists have been proposed as therapeutic approaches for several inflammatory/autoimmune diseases such as multiple sclerosis, Crohn’s disease, rheumatoid arthritis, pancreatitis, etc. (25, 30–32).

Even though all these evidences support a physiological role of Ghrl in early gestational events such as decidualization, embryo implantation, placentation and immunomodulation, little is known about the putative consequences of Ghrl misbalance on embryo implantation and development. In fact, the scarce reported data mainly comes from *in vitro* studies. However, using our *in vivo* experimental model of gestational Ghrl misbalance, we previously showed that not only hypergrhelinemia, but especially the inhibition of Ghrl activity during early gestation, exerted detrimental effects on fertilization, pre-implantation embryo development and mainly, on implantation. Indeed, administering Ghrl or the antagonist during mice peri-implantation period, significantly higher percentages of atrophied fetuses were found by day 18 of gestation (9). Although these findings strongly suggest that Ghrl misbalance impairs embryo implantation and/or development, the underlying mechanisms remain uncertain.

In the present work, we aimed to evaluate the putative mechanisms underlying those effects. Using our animal model of gestational Ghrl imbalance during the peri-implantation period we analyzed the effects on embryo implantation, uterine NOS activity/NO synthesis and uterine inflammatory/immune profile. Our results show that the inhibition of Ghrl activity by an antagonist during that gestational period induces pro-inflammatory changes and nitrosative stress in the gravid uterus, impairing significantly embryo implantation and/or development, and increasing consequently embryo loss.

## 2 Materials and methods

### 2.1 Animals

Inbreed Albino Swiss (N:NIH) adult mice (60-70 days old) were used. Animals were maintained on a 14:10h light:dark cycle at 23±2°C, and had free access to water and commercial pelleted food (Grupo Pilar-Gepsa, Cordoba, Argentina). Experiments were approved by and conducted in accordance with the Committee for Animal Care and Use of the National University of Cordoba School of Medicine (UNC-RHCS 674/09). A total of 69 animals were used in the study.

### 2.2 Animals’ treatment

Ghrelin (acyl-ghrelin; Pi-Proteomics, Huntsville, USA) and its antagonist (D-Lys_3_)GHRP-6 (Sigma-Aldrich, Schnelldorf, Germany) were both dissolved in isotonic solution (0.9% NaCl solution). As previously described (9, 33, 34), pregnant female mice were treated with subcutaneous injections (0.1ml) of Ghrl (4nmol/animal/day) or (D-Lys_3_)GHRP-6 (6nmol/animal/day) solutions twice a day (at 9:00a.m. and 5:00p.m.; half the daily dose each time). Previous experiments have demonstrated that Ghrl and antagonist doses used in this experimental protocol do not modify significantly either daily food intake or body weight (9); which was corroborated in the present study (data not shown). The dose of Ghrl used was set up based on its ability to increase Ghrl serum levels and growth hormone secretion from a dose-response curve (33, 34). Accordingly, the selected antagonist treatment dose was previously shown to inhibit the effects of either endogenous or exogenously induced hyperghrelinemia (34). Control dams received the vehicle.

Females were mated with males (in a rate 2:1) in the day when the natural proestrous was detected. During the morning of the following day, vaginal smears were analyzed and copulation was considered positive when the copulatory plug or spermatozoa were observed. This was considered as day 1 of pregnancy. Dams were treated with Ghrl (Ghrl group), the antagonist (D-Lys_3_)GHRP-6 (Ant group), or vehicle (C group) from day 3 to day 8 of gestation (peri-implantation period). At day 8 of gestation and 30min after the last dose injection, females were weighted and then euthanized by decapitation to analyze the effects on embryo implantation, uterine NOS activity/NO synthesis and uterine inflammatory/immune profile. It is important to highlight that: 1) mouse embryos are supposed to adhere to endometrium between day 4-5 of pregnancy, but penetration (which in mice includes decidualization) lasts to day 8, in which placental growth begins the replacement of the decidual zone (35); 2) implantation depends on several dams´ and embryos´ factors, virtually impossible to separate within the complete implantation process, and 3) embryo development is a continuous process. In consequence, the results found in this study in which Ghrl misbalance is applied from day 3 to 8 of pregnancy, might be attributed particularly to implantation defects, but also to early implanted embryo developmental defects. So in this manuscript we will refer to effects on “implantation and/or embryo development” or “implantation and pregnancy success”, in order to cover both inseparable processes.

### 2.3 Parameters

#### 2.3.1 Samples processing for histopathology and immunohistochemistry analysis

After euthanasia, gravid uteri were exposed, excised, weighted (to calculate relative uterine weight) and fixed in tubes containing buffered formol. Then, the fixed uterine horns were embedded in paraffin and cut into 4μm thick sections obtained every 30μm at three depths. These sections were mounted on positively charged glasses (Biotraza), deparaffinized (with xylene two times for 10min), rehydrated (with decreased graded series of ethanol -for 5min each- and distilled water) and stained with Hematoxylin/Eosin for histopathology, and counterstained with Hematoxylin for immunohistochemistry.

#### 2.3.2 Microscopic evaluation of implantation sites and reabsorbed embryos

The embryos and the points indicative of implantation and/or resorption sites were quantified in both uterine horns. Also, the implantation areas were measured using digitized photos and Axiovision 3.0.6 software (Carl Zeiss Vision, Germany). Results were expressed as mm^2^.

#### 2.3.3 eNOS, iNOS and nitrotyrosine tissue expression analysis

The expression of eNOS, iNOS (inducible NOS) and nitrotyrosine levels were assessed in uterine tissue by immunohistochemistry. For antigen retrieval, slides were treated with sodium citrate buffer (pH 6.0) in a pressure cooker for 20min. Endogenous peroxidases were blocked by incubating slides with 0.3% H_2_O_2_/PBS for 10min. Sections were blocked with PBS containing 20% FCS for 20min at room temperature, and then incubated overnight with a purified rabbit polyclonal antibody (Abcame NOS -ab66127- or anti iNOS -H-174-; Santa Cruz Biotechnology Inc., Santa Cruz, CA, USA). Afterwards, sections were incubated with a biotin-conjugated Multilink secondary antibody and labeled with streptavidin-peroxidase.

For the detection of nitrosylated tyrosines, a polyclonal antibody (sc-32731- Santa Cruz Biotechnology Inc., Santa Cruz, CA, USA) and a Novolink Polymer Detection System (Leica Biosystems) were used. Unspecific antigens were blocked with 0.4% casein in phosphate-buffered saline (pH 7.4). The sections were incubated overnight with nitrotyrosine primary antibody in a wet chamber. Subsequently, sections were incubated with an anti-rabbit mouse IgG (Post Primary) for 30min and finally with Poly-HRP-IgG anti-rabbit (Polymer) for 30min at 37°C.

The binding was detected using Vectastain Elite ABC kits (Vector Laboratories, Burlingame, CA). Product was revealed according to the manufacturers’ instructions kit DAB (Vector Laboratories, Burlingame, CA) using hematoxylin (Biopur) as counterstain. Sections were mounted with glass coverslips using DPX (Sigma Aldrich).

Image analysis was used in order to quantify immunohistochemical expression of eNOS, iNOS and nitrotyrosine. Digital images were captured using Leica DM500 biological microscope (DM500, Leica, Wetzlar, Germany) at 40x magnification. The TIFF format images were processed using computer-assisted image analysis Fiji/ImageJ software (https://imagej.net/Colour_Deconvolution) (36). Expression was quantified by calculating the % of positive area (µm^2^)/total area (µm^2^) analyzed, in at least 20 random digital images from four-five mice uteri for each experimental condition (37).

#### 2.3.4 Plasma progesterone levels

After euthanasia, blood specimens were collected in tubes previously embedded in EDTA and centrifuged at 120g for 30min to obtain plasma, which was stored at −20°C until analysis. Progesterone plasma concentration was determined with an in-house enzyme immunoassay (EIA) using a polyclonal antibody, progesterone standard and the corresponding horseradish peroxidase conjugate (anti-Progesterone R4859, Department of Population Healthand Reproduction, C. Munro, UC Davis, CA, USA). Briefly, flat-bottom microtiter plates (NuncMaxisorp, VWR, Mississauga, ON, Canada) were coated with 50μl of the antibody diluted in coating buffer (50mM bicarbonate buffer, pH 9.6), covered with acetate sealers to prevent evaporation and incubated overnight at 4°C. After 16-24hr, plates were washed to remove any unbound antibody with 0.02% Tween 20 solution using a Bio-TekELx 405VR microplate washer (Bio-Tek Instruments, Winooski, VT). Immediately after washing, 50μl of plasma samples, standards and controls were added in duplicates, followed by 50μl of horseradish peroxidase conjugate diluted in EIA buffer. Plates were then covered and incubated at room temperature for 2hr. Following incubation, plates were washed and blotted dry, and 100μl of substrate solution [50mM citrate, 1.6mM hydrogen peroxide, and 0.4mM 2,20-azino-di-(3-ethylbenzthiazoline sulfonic acid) diammonium salt, pH4.0] were added to each well (38). Absorbance was measured at 405nm using a microplate reader (BioTek Instruments, Synergy LX, USA). The sensitivity of the assay for progesterone was 0.018ng/ml and the intra- and inter-assay coefficients of variations were <10%.

#### 2.3.5 Nitric oxide quantitation

Nitric oxide secretion in uterine tissue was assessed by the Griess method. After euthanasia, the entire uteri were excised, weighted and incubated with shaking in tubes containing a proportion of 3:1 media (ml):tissue (g) of RPMI media containing arginine, for 1 hour at 37°C (95% air-5% CO_2_). After incubation, tubes were centrifuged at 40g for 10min and supernatants collected. As previously described (39), the production of NO was measured as nitrites in the supernatants by mixing 50µl of the latter with an equal volume of Griess reagent (Britania). After incubating at room temperature for 10min, the absorbance was read at 540nm. Nitrite concentrations in samples were calculated from a standard curve using NaNO_2_. Results were expressed as µM.

#### 2.3.6 Cytokine expression analysis in uterine tissue

The expression levels of VEGF (vascular endothelial growth factor), interleukin-10 (IL-10), IL-17, IL-6, MMP9 (matrix metalloproteinase 9) and GM-CSF (granulocyte macrophage–colony stimulating factor) were assessed in uterine tissue by quantitative Polymerase Chain Reaction (qPCR) using specific primers as previously described (40). Briefly, after removing the embryos, entire uteri were conserved in RNA later till processing (41). Total RNA of each uterus was then extracted using the TRIzol method (Life Technologies, Gaithersburg, MD, USA). RNA integrity was determined by direct visualization of samples electrophoresed in 1% agarose gel in TAE buffer (40mM Tris-Acetate, 1mM EDTA). The RNA concentration and purity was determined using a Nanodrop 1000 spectrophotometer (Thermo Scientific). An aliquot of 2μg RNA was treated with 1μl of DNAse (1UI/ul, Invitrogen) for 15min at room temperature and denatured at 65°C for 10min. cDNA was synthesized in a 10μl reaction volume containing DNAse-treated RNA (2μg), 1U RNAse inhibitor (Promega), 2μg of random primers (Biodynamics), 2mM dNTP (Promega) and 10U of M-MLV Reverse Transcriptase (Promega). The reaction mixture was incubated for 1h at 42°C followed by incubation at 65°C for 10min. For qPCR amplification, 5μl of the prepared cDNA (prior diluted 1/5) was added to a tube containing 40μM dNTP; 10μM primer pairs, and SYBR Green PCR Master Mix (Applied Biosystems). The PCR reactions were carried out using Step One Plus real time PCR equipment (Applied Biosystems) with the following cycling condition: 10min at 95°C, followed by 40 cycles of 95°C for 15s and 60°C for 1min. Comparative CT (ΔΔCt) was use as quantitation method. *Eef2* amplification was used for data normalization. The sequences of the specific primers used are listed in **Supplementary Table 1**.

#### 2.3.7 Analysis of uterine tissue infiltrating leukocytes

Uterus-infiltrating leukocyte analysis was performed by flow cytometry as previously described (40). After embryo removal, freshly excised entire uteri from control and treated mice were mechanically disrupted and enzymatically digested in RPMI 1640 medium containing 1mg/ml collagenase D (Roche, Basilea, Switzerland) and DNase I (Sigma-Aldrich) for 45min at 37°C (42). After digestion, suspensions were filtered through 75- and 40-μm cell strainers (BD Biosciences) and single-cell suspensions were washed twice in RPMI 1640 with 10% FBS, 2mM EDTA, and 50mM 2-ME supplemented medium. Live lymphocyte counts were deduced from the acquisition of a fixed number of 10-mm latex beads (Beckman Coulter, Brea, CA) mixed with a known volume of unstained cell suspension in propidium iodide (BD Biosciences). Analyses were performed on a FACS Canto II using DIVA software (BD Biosciences), allowing the exclusion of dead cells (propidium iodide positive) inside the indicated gates. After that, cells were stained with different fluorescent-labeled specific antibodies for FACS analysis: anti-mouse CD3 to identify T lymphocytes, anti-mouse NK1.1 for NK cells, anti-mouse Gr1 for granulocytes, anti-mouse CD11c/CD11b for dendritic cells, and anti-mouse CD11b/Gr1 for macrophages. At least 200,000 events/cells per specimen were acquired using a FACS Canto II, and data were analyzed using FlowJo software (Tree Star).

### 2.4 Statistics

Data were analyzed using Infostat 2020 statistical software (Infostat Group, Facultad de Ciencias Agropecuarias – Universidad Nacional de Córdoba, Argentina). Results were expressed as Mean ± SEM and analyzed using a one-way ANOVA with LSD Fisher as *post-hoc* comparison analysis. To apply this test, variance homogeneity and Gaussian distribution were confirmed. Significance level was set at 0.05.

## 3 Results

### 3.1 Ghrelin inhibition during the peri-implantation period impairs pregnancy outcome

Mice dams treated with 6nmol/animal/day with the Ant (D-Lys3)GHRP-6 from day 3 to 8 of pregnancy evidenced impaired pregnancy outcomes with respect to Ghrl-treated or C mice, since the former showed significantly higher rates of embryo resorption (**Table 1**). Conversely, the hyperghrelinemia secondary to Ghrl-treatment did not affect pregnancy outcome since results were comparable to C on implantation sites and reabsorbed embryos (**Table 1**). Besides, no significant differences were observed among groups when analyzing dams´ body weight, relative uterine weight, mean implantation area, or plasma progesterone (**Table 1**).

**Table 1:** Effects of ghrelin or its antagonist at peri-implantation period on embryo implantation and pregnancy success.

Parameter	C	Ghrl (4 nmol/animal/day)	Ant (6 nmol/animal/day)
Dams body weight (g)	30.4 ± 1.3	30.5 ± 1.1	30.5 ± 1.3
Relative uterine weight (%)	1.5 ± 0.1	1.3 ± 0.1	1.2 ± 0.1
Total implantation sites	13.0 ± 0.9	13.2 ± 0.8	12.2 ± 0.9
Reabsorbed embryos (%)	4.4 ± 4.0	8.9 ± 3.6	30.2 ± 4.0 *
Implantation area (mm^2^)	4.1 ± 0.5	3.7 ± 0.4	3.0 ± 0.5
Plasma progesterone (ng/ml)	6.0 ± 1.0	7.0 ± 0.7	7.0 ± 0.8

Copula was confirmed by vaginal plug or spermatozoa in vaginal smears after mating assays. Ghrelin (Ghrl) or its antagonist [Ant: (D-Lys_3_)GHRP-6] were s.c. injected from gestational day 3 to 8. Control (C) dams were injected with the vehicle (0.9% NaCl) in the same regime. Dams were euthanized at gestation day 8, 30min after the last injection. Uteri were excised and the number of implantation sites and the efficiency of implantation (viable or reabsorbed embryos) were microscopically recorded. Results are expressed as Mean ± SEM and were analyzed by one-way ANOVA/LSD Fisher as post-hoc comparison. n= 6-7 dams/treatment. *: p< 0.01 vs. control and ghrelin.

### 3.2 Ghrelin misbalance induces uterine nitrosative stress

**Figure 1:**
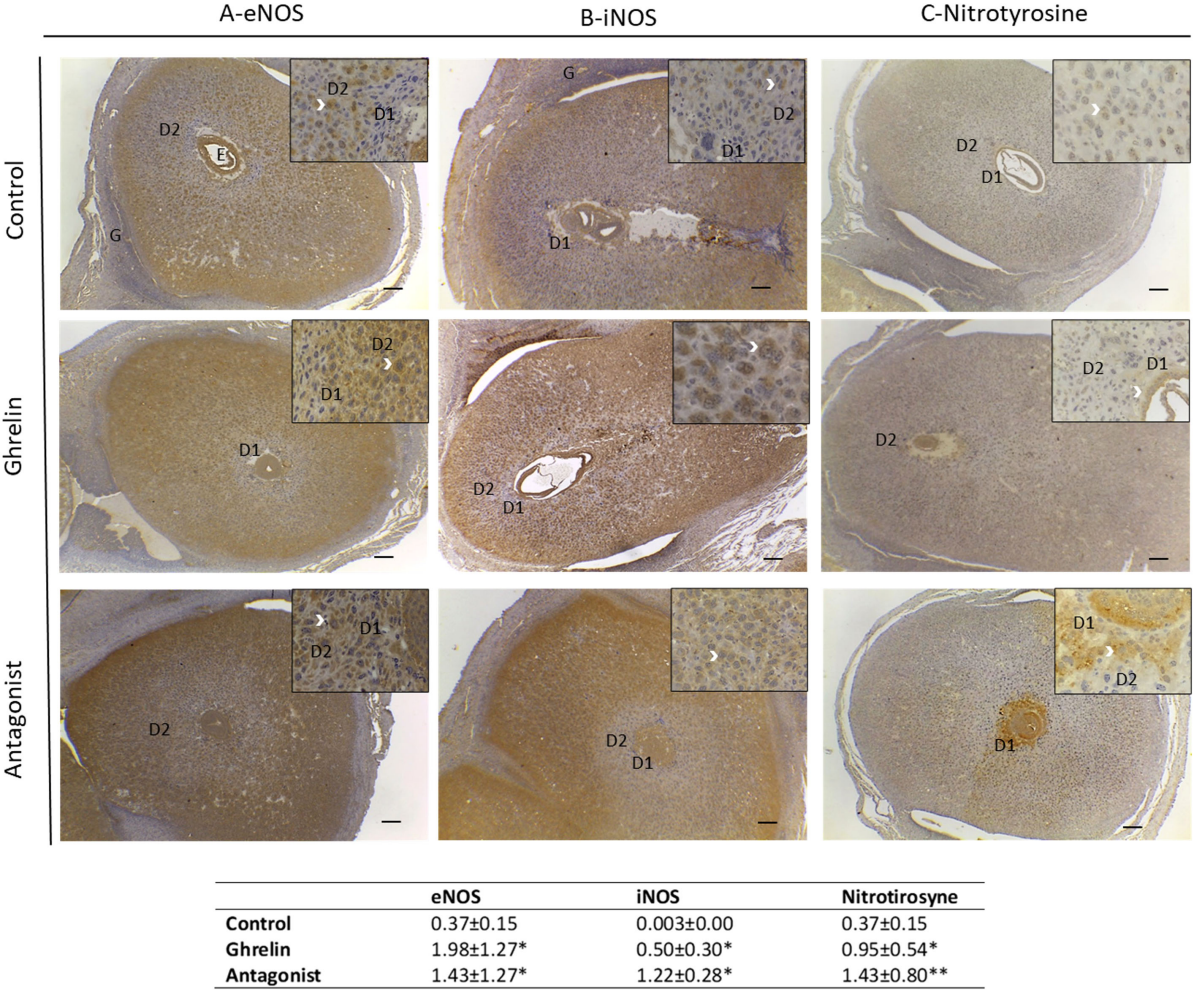
Expression of eNOS **(A)**, iNOS **(B)** and nitrotyrosine **(C)** in uterine tissue and quantification of this expression (table below the images) by calculating the % of positive area (µm^2^)/total area (µm^2^) analyzed. Uteri were obtained from dams treated with ghrelin (Ghr: 4 nmol/animal/day), its antagonist [Ant: (D-Lys3)GHRP-6; 6 nmol/animal/day] or vehicle (C: control), from gestation day 3 to 8. Dams were euthanized at day 8 and uteri exposed and fixed for immunohistochemistry. Number of females/group=4-6. Scale bar 50 μm. E=embryo; D1=primary decidual zone; D2=secondary decidual zone and G=glandular epithelium. White arrows indicate immunostaining. Data were analyzed by one-way ANOVA/LSD Fisher as *post-hoc* comparison. *:*p*<0.05 vs. control; **:*p*<0.001 vs. control and ghrelin.

When assessing the effect of Ghrl or Ant on eNOS and iNOS expression in uterine tissue (**Figures 1A, B**), we found that both treatments significantly increased the expression of both enzymes, especially that of iNOS, which showed more than 350 fold increase with respect to C.

Moreover, Ghrl or Ant treatments resulted in different expression pattern of eNOS and iNOS.

Regarding eNOS, in C animals it showed to be expressed mainly in the embryo, the secondary decidual zona, glandular epithelium and blood vessels. In the cases of Ghrl- or Ant-treated animals, eNOS expression was mainly observed in the decidual zone, including the primary and secondary zone of decidualization (**Figure 1**). With respect to iNOS, C animals showed iNOS to be expressed mostly in the secondary decidual zone, on the periphery of the implantation site, and in some cells of the glandular epithelium; whereas Ghrl-treated animals showed iNOS expression mostly in the embryo and in some stromal cells (**Figure 1**). Conversely, Ant-treated animals evidenced a decreased iNOS expression in the embryo, but increased in stromal cells (**Figure 1**).

**Figure 2:**
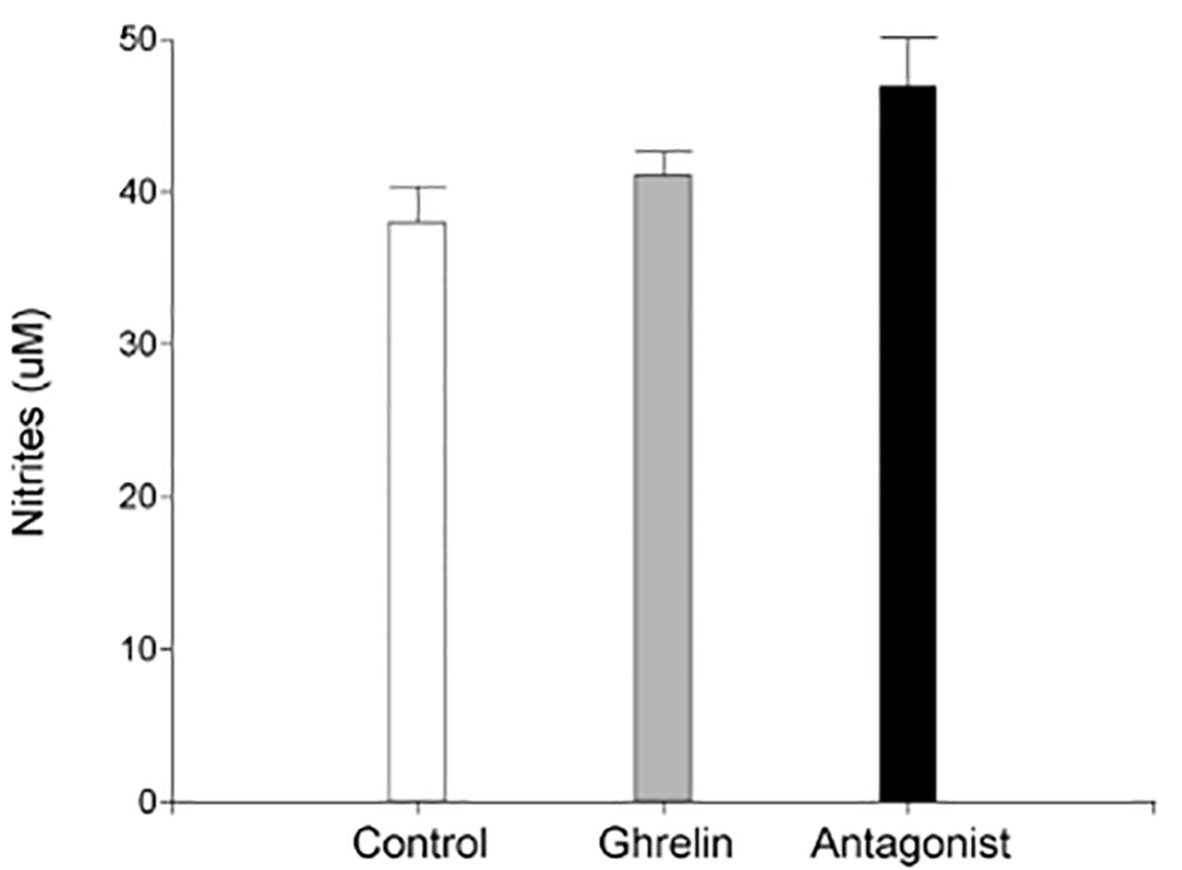
Uterine nitric oxide production. Uteri were obtained from dams treated with ghrelin (Ghr: 4 nmol/animal/day), its antagonist [Ant: (D-Lys3)GHRP-6; 6 nmol/animal/day] or vehicle (C: control), from gestation day 3 to 8. Dams were euthanized at day 8 and uteri exposed and collected for incubation. Nitrites were evaluated by Griess method. Results are expressed as concentration (μM, Mean ± SEM) and were analyzed by one-way ANOVA/LSD Fisher as *post-hoc* comparison. Number of females/group=5-6. No significant differences among treatments.

Based on these findings, we subsequently evaluated uterine NO secretion. As shown in **Figure 2**, no significant differences were found in nitrite levels among the different experimental groups under study. Afterwards, we evaluated protein tyrosine nitration levels in uterine tissue. Since protein tyrosine nitration is mediated by reactive nitrogen species such as peroxynitrite anion and nitrogen dioxide, it is indicative of nitrosative stress (43). In our study, we found significantly higher nitrotyrosine expression levels in uterine tissue from Ghrl- and Ant-treated animals than in the C ones (**Figure 1C**).

Moreover, nitrotyrosine expression in control animals showed to be evident only in a few cells of the decidual stroma (**Figure 1**). In turn, its expression in Ghrl-treated animals was restricted to some cells of the embryo and the primary zone of decidualization. Ant-treated animals showed increased nitrotyrosine expression in the embryo, primarydecidualization zone, and in all the decidualization stroma (**Figure 1**). These results indicated that a Ghrl misbalance results in nitrosative stress in the decidualization zone which might impair implantation and pregnancy success.

### 3.3 Ghrelin misbalance induces an uterine dysregulated immune/inflammatory profile

**Figure 3:**
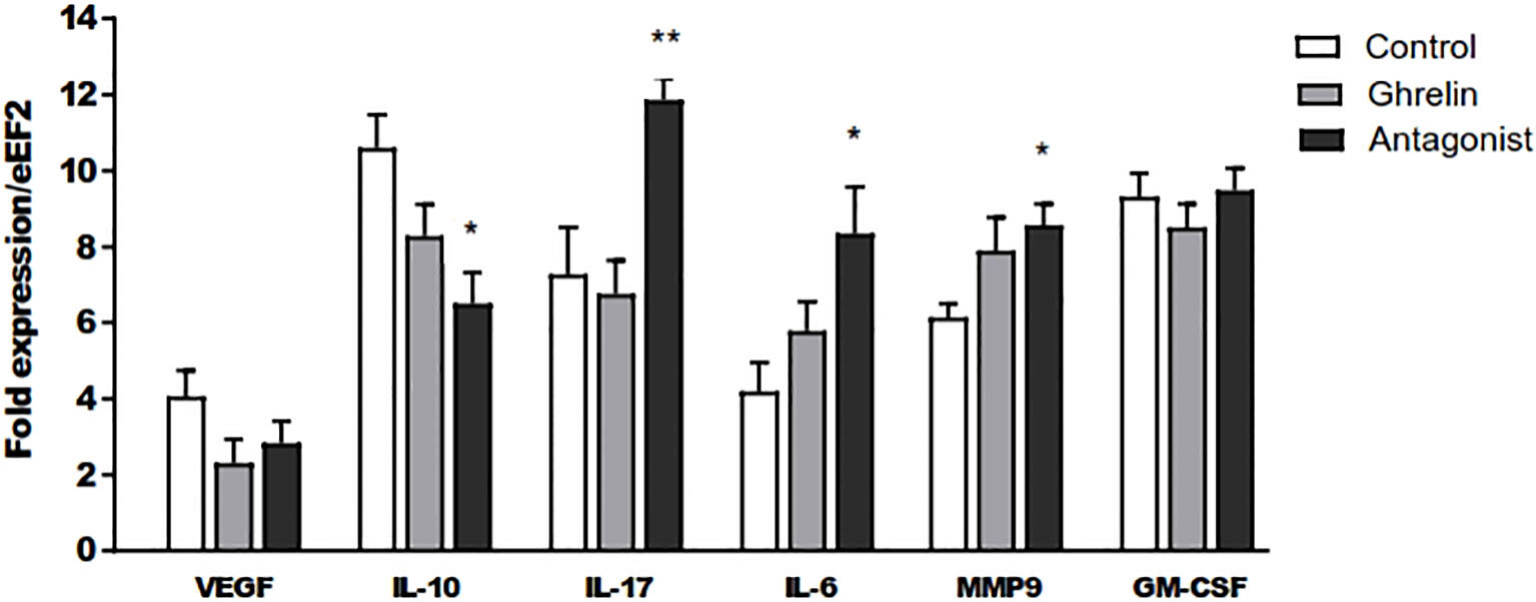
Relative expression of IL-10, VEGF, IL-17, IL-6, MMP 9 and GM-CSF to the eEF2 housekeeping gene assayed by qPCR in uterine tissue. Dams were treated with ghrelin (Ghr: 4 nmol/animal/day), its antagonist [Ant: (D-Lys3)GHRP-6; 6 nmol/animal/day] or vehicle (C: control), from gestation Day 3 to 8. Dams were euthanized at Day 8, uteri exposed and collected. Total mRNA was purified by extraction columns and qPCR run following routine standard protocols in our laboratory. Results are expressed as Mean ± SEM and were analyzed by one-way ANOVA/LSD Fisher as *post-hoc* comparison. Number of females/group=5-6. *:*p*<0.05 vs. control; **:*p*<0.05 vs. control and ghrelin.

To assess if Ghrl misbalance in the peri-implantation period affects the expression of key factors involved in decidualization, embryo implantation and development, the mRNA expression of different cytokines, growth factors and MMP9 was assessed by qPCR in uterine tissue at day 8 of gestation. As shown in **Figure 3**, the treatment with (D-Lys3)GHRP-6 resulted in significantly enhanced expression of the MMP9 and the pro-inflammatory cytokines IL-17 and IL-6, and reduced expression levels of the immunoregulatory cytokine IL-10, in comparison to C animals (**Figure 3**). A similar trend was observed in Ghrl-treated animals; however, they did not reach statistical significance (**Figure 3**). On the other hand, no significant differences were detected among groups, in the uterine expression of VEGF or GM-CSF (**Figure 3**).

**Figure 4:**
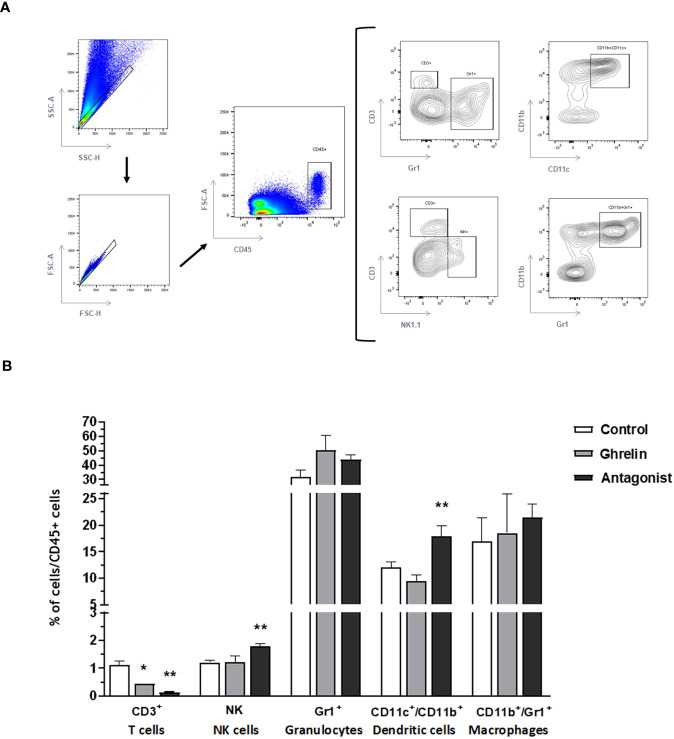
Analysis of uterine infiltrating leukocytes by flow cytometry. Gating strategies **(A)** and frequencies of uterine tissue infiltrating leukocyte subsets **(B)**: T lymphocytes(CD3^+^), NK cells (NK1.1), granulocytes (Gr1^+^), dendritic cells (CD11c^+^/CD11b^+^), and macrophages (CD11b^+^/Gr1^+^). Dams were treated with ghrelin (Ghr: 4 nmol/animal/day), its antagonist [Ant: (D-Lys3)GHRP-6; 6 nmol/animal/day] or vehicle (C: control), from gestation Day 3 to 8. Results are expressed as frequencies of CD45+ cells (leukocytes), Mean ± SEM, and were analyzed by one-way ANOVA/LSD Fisher as *post-hoc* comparison. Number of females/group=5-6. *:*p*<0.05 vs. control; **:*p*<0.05 vs. control and ghrelin.

In parallel, uterine-infiltrating leukocyte subsets were analyzed. Interestingly, Ant-treated animals showed significantly higher frequencies of NK cells and dendritic cells (the subtype CD11b^+^) than C animals (**Figure 4**). Strikingly, either Ant- or Ghrl-treated animals showed significantly reduced frequencies of infiltrating T cells with respect to C animals (**Figure 4**). No differences were detected in the frequencies of infiltrating granulocytes or macrophages among groups under study.

Altogether, these results indicate that Ghrl misbalance during the peri-implantation period induces immune changes in the uterus associated to inflammation which might impact on implantation and pregnancy success.

## 4 Discussion

The main objective of our study was to assess the physiological role of Ghrl on embryo implantation and/or development using a validated animal model of gestational Ghrl misbalance. Our results support the notion that Ghrl is a key factor necessary for successful embryo implantation and pregnancy success, since the inhibition of its activity by an antagonist during the peri-implantation period significantly impairs embryo implantation, and consequently increases embryo loss. At least some of the mechanisms underlying this effect involve the induction of pro-inflammatory changes and nitrosative stress in the early gravid uterus, since hyperghrelinemia or Ghrl-blockade by antagonist increased eNOS/iNOS and nitrotyrosine expression and induced pro-inflammatory cytokine expression, altering also leukocyte infiltration. In fact, the treatment with the Ghrl antagonist (D-Lys3)GHRP-6 exerted pro-inflammatory changes in the early gravid uterus that are hostile to implantation such as increased expression of IL-6, IL-17 and MMP9, augmented infiltration of NK and dendritic cells (type CD11b^+^) cells, and decreased infiltration of T cells and reduced expression of the immunoregulatory cytokine IL-10. In addition, the hyperghrelinemia achieved with the Ghrl treatment dose used in this study showed a similar pattern, although with less marked effects.

Ghrelin has been proposed as one of the multiple factors that promote uterine remodeling for embryo implantation. It has been shown that Ghrl and its cognate receptor (GHS-R1a) are expressed in human endometrium, with higher expression levels at glandular epithelium and stromal cells of the secretory phase, when the endometrium becomes receptive to embryo implantation (7). The fact that both, Ghrl and GHS-R1aare expressed in the same tissues, suggests autocrine/paracrine actions of Ghrl (7). *In vitro* experiments have also revealed that Ghrl promotes decidualization since, when combined with progesterone, the peptide enhances the production of prolactin and IGFBP-1 by human endometrial stromal cells (7). Similar results were reported by Tanaka et al. (2003), whose data showed a dramatic increase of Ghrl expression in human first trimester deciduas and *in vitro* decidualization-stimulating effects of the peptide on endometrial stromal cells (8). Furthermore, Ghrl has been also shown to stimulate the placental JEG-3 cell proliferation and made them resistant to apoptosis (15). All these evidences support the hypothesis that the distinctive increase on Ghrl secretion observed at early pregnancy (3–5) contributes to endometrial decidualization and placenta formation. In fact, an “ideal” Ghrl level for optimal embryo development has already been reported (9–11). Therefore, it is possible that alterations in this expected Ghrl raise exert deleterious effects on embryo implantation. Or, more importantly, that alterations in plasma Ghrl concentrations, as commonly observed in diseases associated to subfertility like polycyctic ovarian syndrome or obesity (44, 45), might impair female fertility.

Our results showed that the administration of a Ghrl antagonist [(D-Lys3)GHRP-6, at 6 nmol/animal/day] during the peri-implantation window, significantly impaired embryo implantation and/or embryo development, since it induced a more than 6-fold increase in the rate of reabsorbed embryos. This is consistent with reported evidence indicating an *in vitro* stimulating effect of Ghrl on endothelial cell proliferation and angiogenesis, which is crucial for embryo implantation and development (17).

Moreover, pregnant hyperghrelinemic females (subjected to a Ghrl treatment) showed a two-fold increase in the percentage of embryo resorption with respect to pregnant normoghrelinemic controls. Although this difference did not reach statistical significance, the observed trend suggests that, as observed for the loss of Ghrl function, hyperghrelinemia may also affect implantation and pregnancy success. It is important to remark that when the same dose of Ghrl was applied for the same period, but results evaluated at pregnancy day 18, we found for Ghrl a significant deleterious effect on post-implantation loss (9). This make us wonder if the effects of Ghrl, that in the current study were less important than that of the Ant, would be statistically evident only with pregnancy ongoing.

Despite increased embryo resorption/loss, the treatments in our model did not alter other parameters like dam´s weight, relative uterine weight, total implantation sites or implantation area. Moreover, alterations in embryo implantation and/or development were not related to progesterone secretion, since dam´s plasma progesterone levels were comparable in the three experimental groups. These findings indicate that factors altering Ghrl levels during early pregnancy would be particularly detrimental for implantation and/or embryo development. Noteworthy, Ghrl is a gut hormonal peptide that functions as a starvation signal (1, 2); thus, when increased it may inhibit reproductive process in order to prioritize life during low energy scenarios (12, 13). Besides, it is important to highlight that the treatment dose of Ghrl used in this study, although enough to stimulate growth hormone secretion, is not high enough to significantly increase food intake (9, 33). Thus, although it seems to be a low dose to be considered as a starvation signal, it still exerted an effect upon embryo implantation. In consequence, more research conducting *in vivo* studies and assessing different Ghrl regime doses need to be performed in order to ascertain the precise effects of hyperghrelinemia on embryo implantation and development. For example and as mentioned above, Sabatini et al. (2009) found that underweight patients undergoing assisted reproduction, showed worse results in clinical pregnancy rate and lower concentrations of plasma estradiol and progesterone and they showed also a fivefold increase in ghrelinemia secondary to underweight (14). On the contrary, in the *in vivo* model used in this study, the administration of Ghrl to pregnant mice exerted only a 2.7 fold increase in ghrelinemia; i.e. lower than that seen in Sabatini´s work. This reinforces the necessity of assessing higher Ghrl doses in order to mimic undernourishment and to evaluate their possible negative effects on reproductive outcome. Anyway, the degree of ghrelinemia increase provoked by exogenous Ghrl administration seen in this study is comparable with that observed previously in 50% food restricted female mice (46).

In order to explore the mechanisms underlying the increased embryo resorption observed with Ghrl misbalance, we then analyzed if our protocol modified uterine NO production and/or induced nitrosative stress, since stimulating effects of Ghrl on NO synthesis has already been reported (18, 19). Results indicated that not only Ghrl but also the Ant treatment significantly increased eNOS and iNOS expression, being the latter especially up regulated. These results are in agreement with previously reported evidence from non-reproductive experimental models showing that Ghrl stimulates NOS activity (18–20). Furthermore, cultured endothelial cells exposed to Ghrl showed increased eNOS activity in a dose-dependent manner (47). Nevertheless, up to our knowledge, there is no reported data from the assessment of the effects of Ghrl blockade on NOS expression.

It is well accepted that low NO concentrations produced by eNOS are involved in maintaining basic physiological functions such as vascular homeostasis and neutrotransmission, and generally exhibit anti-inflammatory effects. Conversely, high concentrations of NO, i.e. those iNOS derived, mediate pro-inflammatory responses, being involved in extracellular matrix remodeling, induction of apoptosis and tissue destruction (48). Hence, we speculated that the increase in NOS expression, especially that of iNOS, would greatly enhance NO synthesis. To prove that, we assessed NO production and nitrosative stress in day 8 gestation uterine tissue from dams treated with Ghrl or Ant. Intriguingly, although nitrite levels were increased in uterine tissue fromGhrl- and Ant-treated mice (Ant>Ghrl>C), the observed differences did not reach statistically significance (p=0.072). However, significantly higher levels of nitrotyrosine were detected in uterine tissue from Ghrl-and Ant-treated females indicating nitrosative stress-associated tissue damage, what could be explained by the metabolization of the excessive nitrites to peroxinitrites.

A fundamental issue in reproductive immunology is that successful reproduction relies on a precise regulation of the immune response during pregnancy, which requires limited inflammation during implantation, the induction of immune tolerance to alloantigens during early and mid-pregnancy, and the trigger of a pro-inflammatory response early before parturition (reviewed in 49 and 50). In fact, the early induction of regulatory T cells and their recruitment to decidua have been shown to be key for successful pregnancy development. On the contrary, dysregulated cytokine production and altered immune cell recruitment and infiltration associated to inflammation are detrimental for embryo implantation and growth, usually leading to pregnancy disorders such as pre-eclampsia and miscarriage (49–51). This abnormal endometrial immune profile is characterized by increased levels of pro-inflammatory cytokines, such as IL-17, IFNγ and TNF-α, secondary to an impaired balance between pro-inflammatory effector immune cells and regulatory T cells (52). Considering that, and taking into account the immunomodulatory effects reported for Ghrl, we then aimed to evaluate if the deleterious effects on pregnancy outcome induced by Ghrl misbalance were associated with immune alterations in uterine tissue. Indeed, the blockade of Ghrl during pre-implantation, achieved by the treatment with the Ant (D-Lys3)GHRP-6, significantly increased uterine expression of the pro-inflammatory cytokines IL-6 and IL-17, which are embryotoxic and exert anti-trophoblast activities when increased, thus inducing pre-eclampsia and recurrent miscarriage (53, 54). Concordantly, the Ant-treatment also decreased the expression of IL-10, a key immunoregulatory cytokine involved in Treg function and embryo receptivity (49, 55). Besides, a similar trend in the pattern of cytokine expression was found in uterine tissue from Ghrl-treated dams, supporting the notion that Ghrl misbalance during the peri-implantation window impairs the induction of immunoregulation necessary for embryo implantation and development.

Furthermore, and in consonance with results discussed above, the assayed Ant-treatment induced increased expression of MMP9 in the uterus. As other matrix metalloproteinases, MMP9 is capable of digesting components of the extracellular matrix, thus regulating tissue remodeling. In fact, MMP-9 is regarded to be a key enzyme during implantation, as an important regulator of vascular and uterine remodeling in healthy pregnancy (56). Also, dysregulation of MMP-9 has been implicated in abnormal vasodilation, placentation, and uterine expansion in preeclampsia (56–58). Interestingly, it has been shown that, in inflammatory scenarios such as the one found in our study, the upregulation of NO secretion or nitrosative stress can induce MMP9 expression in a dose-dependent manner (59, 60). Furthermore, it has been shown that migrating trophoblast cells express MMP9, which is regulated by NO. Indeed, motile trophoblast cells actively redistribute iNOS to the leading migrating pole of the cell and MMP9 co-localizes with iNOS at the lamellopodia, a phenomenon that seems to be crucial for cell invasion (61). So, in situations of nitrosative stress, the overexpression of MMP9 could impair embryo implantation and vascular remodeling.

Our results also revealed that Ghrl misbalance during the peri-implantation period disturbed the balance of immune cells infiltrating the uterus. In fact, the blockade of Ghrl by the administration of the Ant treatment significantly increased the proportion of infiltrating NK cells and dendritic cells (type CD11b^+^). These results support previously reported evidence showing significantly increased levels of granulysin-positive and CD56^bright^ NK cells in deciduas from women with recurrent spontaneous abortion (62). In fact, increased cytotoxic NK and Th17 cells, and impaired induction of Tregs, are the current hallmark of recurrent miscarriage and pre-eclampsia (63–65). Indeed, women with recurrent spontaneous miscarriage show elevated Th17 responses, a decidual IL-10 deficiency, and extensive local inflammation (66–68). In addition, in two publications Wang et al. (2010) reported increased Th17/Tregs cell ratios associated with unexplained recurrent miscarriage (69, 70). Furthermore, higher expression levels of ROR-γ expression (the Th17 transcription factor) have been detected in implantation sites from reabsorbed fetuses with respect to those from live fetuses in a mouse model of spontaneous miscarriage (71).

Moreover, our results showed significantly increased frequencies of CD11c^+^CD11b^+^dendritic cells, which is a cell subset know to efficiently prime and induce pro-inflammatory Th1 responses (72–74). Intriguingly, our results also showed that both, Ant- and Ghrl-treatment significantly decreased uterus-infiltrating CD3^+^T cells. Although we did not characterized in detail the phenotype of these cells in order to distinguish between effector T cells or Tregs, it has been shown that the vast majority of T cells recruited to the uterus during the peri-implantation period are Tregs (75–77). In consequence, the observed reduction of infiltrating T cells might be due to impaired Treg induction, consequent of Ghrl misbalance. Supporting that, Xia et al. (2004) reported that Ghrl reduced splenic T cell proliferation and decreased secretion of Th1- and Th2-associated cytokines in a dose-dependent fashion (78).

Finally, it is important to mention that the deleterious effects of the Ant-treatment seem not to be related with *per se* effects of the substance. In some of the experiments performed in this study, we evaluated the effects of a fourth experimental group; i.e. Ghrl+Ant. In all the performed assays (embryo implantation, NOS expression and NO secretion in culture assessments) the combined treatment with Ghrl plus the Ant shifted the results to values similar to those of the control group (results not shown).

In conclusion, our results suggest that successfully embryo implantation and/or development requires physiologically balanced Ghrl levels, since the moderate increase of ghrelinemia and especially the inhibition of its function, significantly impairs embryo implantation and pregnancy success. Moreover, the underlying mechanisms seem to involve uterine immune dysregulation and nitrosative stress.

## Data availability statement

The raw data supporting the conclusions of this article will be made available by the authors, without undue reservation.

## Ethics statement

The animal study was approved by Comité Institucional para el Cuidado y Uso de Animales de Laboratorio de las Facultades de Ciencias Médicas y Odontología de la Universidad Nacional de Córdoba. The study was conducted in accordance with the local legislation and institutional requirements.

## Author contributions

EL: Investigation, Methodology, Writing – review & editing. CD-L: Formal Analysis, Investigation, Methodology, Supervision, Writing – review & editing. DP: Formal Analysis, Investigation, Methodology, Writing – review & editing. ND: Investigation, Methodology, Writing – review & editing. PT: Investigation, Methodology, Writing – review & editing. VC: Formal Analysis, Investigation, Methodology, Writing – review & editing. RF: Conceptualization, Formal Analysis, Supervision, Writing – review & editing. RM: Conceptualization, Formal Analysis, Funding acquisition, Project administration, Supervision, Writing – original draft, Writing – review & editing. AM: Conceptualization, Data curation, Formal Analysis, Funding acquisition, Investigation, Project administration, Supervision, Writing – original draft, Writing – review & editing.
