# Supplementary material for: Ghrelin misbalance affects mice embryo implantation and pregnancy success by uterine immune dysregulation and nitrosative stress
Source: Front Endocrinol (Lausanne). 2023 Dec 1;14:1288779. doi: 10.3389/fendo.2023.1288779 (PMC10722256; doi:10.3389/fendo.2023.1288779)
Supplement: Supplementary file 1 [file Table_1.docx]

**Supplementary Table 1:** Sequences of the specific primers used for cytokine expression analysis in uterine tissue

| **Gene** | **Forward primer** | **Reverse primer** |
| --- | --- | --- |
| eEF2 | TGTCAGTCATCGCCCATGTG | CATCCTTGCGAGTGTCAGTGA |
| IL-10 | GGTTGCCAAGCCTTATCGGA | ACCTGCTCCACTGCCTTGCT |
| IL-6 | GAGGATACCACTCCCAACAGACC | AAGTGCATCATCGTTGTTCATACA |
| IL-17 | GCAAGAGATCCGGTCCTGA | AGCATCTTCTCGACCCTGAA |
| VEGF | ACATTGGCTCACTTCCAGAAACAC | GGTTGGAACCGGCATCTTTATC |
| GM-CSF | ACCACCTATGCGGATTTCAT | TCATTACGCAGGCACAAAAG |
| MMP-9 | 5’- CAGAC CAAGGGTACAGCCTGTT - 3’ | 5’- AGTGCATGGCCGAACTC - 3 |
